# PBMC of Multiple Sclerosis Patients Show Deregulation of OPA1 Processing Associated with Increased ROS and PHB2 Protein Levels

**DOI:** 10.3390/biomedicines8040085

**Published:** 2020-04-11

**Authors:** Domenico De Rasmo, Anna Ferretta, Silvia Russo, Maddalena Ruggieri, Piergiorgio Lasorella, Damiano Paolicelli, Maria Trojano, Anna Signorile

**Affiliations:** 1 CNR-Institute of Biomembranes, Bioenergetics and Molecular Biotechnologies, 70126 Bari, Italy; 2 Department of Basic Medical Sciences, Neurosciences and Sense Organs, University of Bari “Aldo Moro”, 70124 Bari, Italy; anna.ferretta@uniba.it (A.F.); silvia.russo92@gmail.com (S.R.); maddalena.ruggieri@uniba.it (M.R.); p.lasorella@studenti.uniba.it (P.L.); damiano.paolicelli@uniba.it (D.P.); maria.trojano@uniba.it (M.T.)

**Keywords:** multiple sclerosis, PBMCs, mitochondria, ROS, OPA1, PHB2, OMA1

## Abstract

Multiple sclerosis (MS) is an autoimmune disease in which activated lymphocytes affect the central nervous system. Increase of reactive oxygen species (ROS), impairment of mitochondria-mediated apoptosis and mitochondrial alterations have been reported in peripheral lymphocytes of MS patients. Mitochondria-mediated apoptosis is regulated by several mechanisms and proteins. Among others, optic atrophy 1 (OPA1) protein plays a key role in the regulating mitochondrial dynamics, cristae architecture and release of pro-apoptotic factors. Very interesting, mutations in OPA1 gene, have been associated with multiple sclerosis-like disorder. We have analyzed OPA1 and some factors involved in its regulation. Fifteen patients with MS and fifteen healthy control subjects (HC) were enrolled into the study and peripheral blood mononuclear cells (PBMCs) were isolated. H_2_O_2_ level was measured spectrofluorimetrically, OPA1, PHB2, SIRT3, and OMA1 were analyzed by western blotting. Statistical analysis was performed using Student’s *t*-test. The results showed that PBMC of MS patients were characterized by a deregulation of OPA1 processing associated with increased H_2_O_2_ production, inactivation of OMA1 and increase of PHB2 protein level. The presented data suggest that the alteration of PHB2, OMA1, and OPA1 processing could be involved in resistance towards apoptosis. These molecular parameters could also be useful to assess disease activity.

## 1. Introduction

Multiple sclerosis (MS) is a complex neurodegenerative disease that involves immune and central nervous system (CNS) [1,2]. MS is expressed in different clinical forms including primary progressive (PP), secondary progressive (SP), progressive relapsing (RP) and relapsing-remitting (RR), which is the most prevalent form [3]. The pathogenesis of MS involves the loss of blood–brain barrier integrity with the consequent invasion of lymphocytes into the CNS resulting in tissue damage [4].

Despite the knowledge of genetics, cell biology and immunology, obtained in the last years, the ultimate etiology or specific elements that trigger MS remain unknown. The etiopathogenesis and pathophysiology of MS involves different factors, among others mitochondrial dysfunction and oxidative stress (OS) play a key role and have a further modulatory effect on many aspects of the disease. OS plays an important role in activation of immune cells, especially T cells [5,6,7], and recently it has been reported that peripheral blood mononuclear cells (PBMCs) of MS patients show impaired redox status associated with mitochondrial alterations [5]. A number of mechanisms participate in the maintenance of the immune homeostasis avoiding the development of autoimmune diseases. The apoptosis is an important anti-autoimmune process that deletes potentially pathogenic autoreactive lymphocytes, limiting the immune response-dependent tissue damage [8,9]. It has been shown that deletion of autoreactive lymphocytes by apoptosis is defective in patients with MS, thereby permitting these cells to perpetuate a continuous cycle of inflammation within the CNS [10,11]. In particular, the impairment of mitochondria-mediated apoptosis in Cd4+ T lymphocytes [12], as well as, a reduction of mitochondrial respiration are reported in MS patients [13]. Mitochondria have a main role in both cell death and life and they are a major source of reactive oxygen species (ROS) production. At the same time, mitochondria are responsive to OS and are critical in modulating apoptosis in response themselves to a variety of stress signals. 

Several mitochondria parameters such as mitochondrial respiratory chain activity, ROS production, dynamics (fusion and fission), and mitochondria cristae architecture are involved in mitochondria-mediated apoptosis [14,15,16]. Among mitochondrial proteins involved in apoptosis mechanism, optic atrophy 1 (OPA1) is a mitochondrial dynamin like GTPase that has attracted great attention for its role in the regulating mitochondrial fusion and fission, the stability of the mitochondrial respiratory chain complexes, pro-apoptotic cytochrome c release and the maintenance of mitochondrial cristae architecture [17]. Very interesting, it is reported that mutations in OPA1 gene, resulting in autosomal dominant optic atrophy (ADOA), are associated with multiple sclerosis-like disorder in patients [18].

OPA1 undergoes constitutive processing leading to the conversion of the un-cleaved long OPA1 (L-OPA1) in a cleaved short OPA1 (S-OPA1) forms. Various stress conditions, including apoptotic stimulation are associated with the conversion of L-OPA1 into S-OPA1. The processing and activity of OPA1 is regulated by mitochondrial proteases, such as OMA1, cellular energetic condition [19], post-translational modification, such as acetylation status [20,21], and oxidative stress [20,22]. OMA1-mediated processing of OPA1 is a cellular stress response, in fact, although OMA1 is constitutively active, it display strongly enhanced activity in response to OS [23]. Furthermore, OPA1 stability is controlled by prohibitin 2 (PHB2) [24] a chaperon like protein, localizes in nucleus, plasma membrane, and mitochondria. Evidences indicate that mitochondrial PHB2 is over expressed under conditions of oxidative stress [25]. Interesting, PHB2 has been found up-regulated in lymphocytes of MS patients [26]. OPA1 processing is also modulated by its acetylation status mediated by SIRT3 enzyme, a mitochondrial deacetylase that also plays an important role in apoptosis [20]. In this work we have analyzed the protein level, and proteolytic processing of OPA1 and its stress-associated regulators, OMA1, SIRT3, and PHB2 in PBMCs of MS patients.

## 2. Experimental Section

### 2.1. Patients

Fifteen patients with MS according to McDonald criteria [27] and fifteen healthy volunteers control subjects (HC) were enrolled into the study. The patients and HC subjects were selected by the Centre of Multiple Sclerosis at Department of Basic Medical Sciences Neurosciences and Sense Organs, University of Bari. All patients had to be without any immunomodulatory treatment at least 6 months prior to study entry. All subjects gave their informed consent for inclusion before they participated in the study. The study was conducted in accordance with the Declaration of Helsinki and the protocol was approved by the Ethics Committee of Azienda Policlinico di Bari (Project identification code 5275). Table 1 reports demographic and clinical characteristics of MS patients and healthy subjects. For all data, no significant difference was observed between males and females as well as between RR and SP forms of MS.

### 2.2. Sample Preparation

Peripheral blood mononuclear cells (PBMCs) were isolated from K3-EDTA blood by centrifugation on a Ficoll-Hypaque density gradient (density: 1.077 g/mL; Amersham Pharmacia Biotech, Buckinghamshire, UK), washed twice and resuspended in PBS. Total protein concentration was determined by Bio Rad protein assay.

### 2.3. Electrophoretic Procedures and Western Blotting

Protein of PBMCs were resuspended in RIPA lysis buffer, separated by 7.5% SDS-polyacrylamide gel electrophoresis (PAGE) and transferred into a nitrocellulose membrane. The membrane was blocked with 5% fatty acid free dry milk in 500 mM NaCl, 0.05% Tween 20, 20 mM Tris, pH 7.4 (TTBS) for 3 h at 4 °C and probed over night with antibodies against OPA1 (whole molecule of OPA1 protein was used as the immunogen) (Thermo scientific, Pierce Antibodies, Lausanne, Switzerland), PHB2 (Invitrogen, Paisley, UK), OMA1, SIRT3 (Cell Signalling, Danvers, MA, USA) and β-actin (Sigma-Aldrich, St. Louis, MO, USA). After being washed in TTBS, the membranes were incubated for 60 min with anti-rabbit or anti-mouse IgG peroxidase-conjugated. Immunodetection was then performed, after further TTBS washes, with the enhanced chemiluminescence (ECL) (Euroclone, Paignton, UK). Densitometric analysis, expressed as arbitrary densitometric units (ADU), electrophoretic profile and relative front (Rf) determinations were performed by Image Lab Touch 2.4 software (BioRad, Milan, Italy). Rf indicates the relative movement of the band from the top.

### 2.4. H_2_O_2_Assay

H_2_O_2_ level was determined by the cell permeant probe 2′-7′-dichlorodihydrofluorescin diacetate (DCFDA). PBMCs were incubated with 10 μM DCFDA in the dark at 37 °C for 20 min, pelleted at 600× *g* for 5 min, washed and resuspended in the assay buffer (100 mM potassium phosphate, pH 7.4, 2 mM MgCl_2_). An aliquot was used for protein determination. The H_2_O_2_ dependent oxidation of the fluorescent probe (507 nm excitation and 530 nm emission wavelengths) was measured by a Jasco FP6200 spectrofluorimeter (Jasco SRL, Cremella, Italy).

### 2.5. Data Analysis

All presented data are means ± standard error of mean (SEM). Statistical difference was determined by Student’ *t*-test. *p*-value of 0.05 was considered as statistically significant (*** *p* < 0.001; ** *p* < 0.01; * *p* < 0.05).

Correlation plots for controls and MS have been performing with Excel Microsoft software using Pearson’s correlation analysis. *P*-values less than 0.05 were considered statistically significant.

## 3. Results

**Figure 1:**
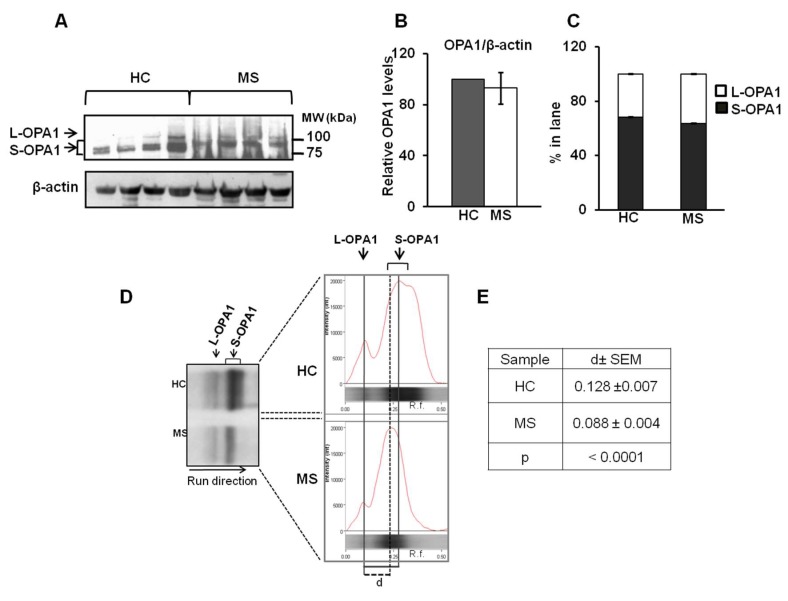
Optic atrophy 1 (OPA1) protein level and processing in peripheral blood mononuclear cells (PBMCs) from HC and MS patients. (**A**) Representative images of western blotting analysis. The PBMC proteins from HC and MS were loaded on 7.5% SDS-PAGE. After separation, the proteins were transferred on nitrocellulose membranes and immunoblotted with the antibody against OPA1. Protein loading was assessed with β-actin antibody. (**B**) The total protein level of OPA1 (L+S forms) was evaluated by densitometric analysis. The arbitrary densitometric units (ADU) of OPA1 were normalized to ADU of β-actin and the mean of HC set to 100%. The histograms represent the percentage values MS patients with respect to HC. The values are means ± SEM of different samples. (**C**) The histograms represent the percentage of ADU of long (L) and short (S), forms of OPA1 in each lane. The values are means ± SEM of samples. (**D**) Left panel, representative image of electrophoretic mobility of immune-revealed bands of OPA1 in PBMCs from HC and MS. Right panel, the images of the western blotting were analyzed by Image Lab Touch 2.4 software (BioRAD) for determination of electrophoretic profile of each lane and calculation of relative front (Rf) of L-OPA1 and S-OPA1 bands (see Table S1). Rf indicates the relative movement of the band from the top of the gel. “d” represents the difference between Rf of S-OPA1 and Rf of L-OPA1 in each lane. (**E**) The table reports the means values of “d” ± SEM of different samples. (*p* < 0.001, Student’s *t*-test).

Fifteen HC and fifteen MS subjects (see Table 1) were enrolled in the study. The patients group included 12 females and 3 males and 11 RR forms and 4 SP forms of MS (see Table 1). The PBMC cellular lysate was used to investigate on the OPA1 protein level and processing by Western blotting analysis using a specific antibody (Figure 1).

The antibody against OPA1 protein, immuno-revealed in both HC and MS groups a long form (L) and short forms (S) of OPA1 (Figure 1A). Densitometric analysis of immuno-revealed bands of OPA1 (L+S) showed the same level of total OPA1protein in MS group with respect to HC (Figure 1B). No difference was observed in percentage of L and S form of OPA1 between HC and SM samples (Figure 1C). Interestingly, the image analysis of western blotting revealed two bands of S-OPA1 in HC and one band of S-OPA1with a different electrophoretic mobility with respect to HC samples in MS (Figure 1A,D). Analysis by Image Lab Touch 2.4 software confirmed the presence of two S-OPA1 bands in HC and one S-OPA1 band in MS as shown by curve peaks (Figure 1D). Moreover, the determination of Rf of L-OPA1 and S-OPA1 bands revealed a significant decrease in Rf of S-OPA1 in MS sample with respect to HC (see Table S1). No differences were observed in Rf of L-OPA1 between HC and MS samples (Table S1). Calculation of difference between Rf of S-OPA1 band and L-OPA1 band in each lane (“d”), revealed a significant decrease of “d” in MS samples with respect to HC (Figure 1D,E). This suggested that processing of OPA1, in MS samples, generated a S-OPA1 form at a higher molecular weight with respect to HC. 

**Figure 2:**
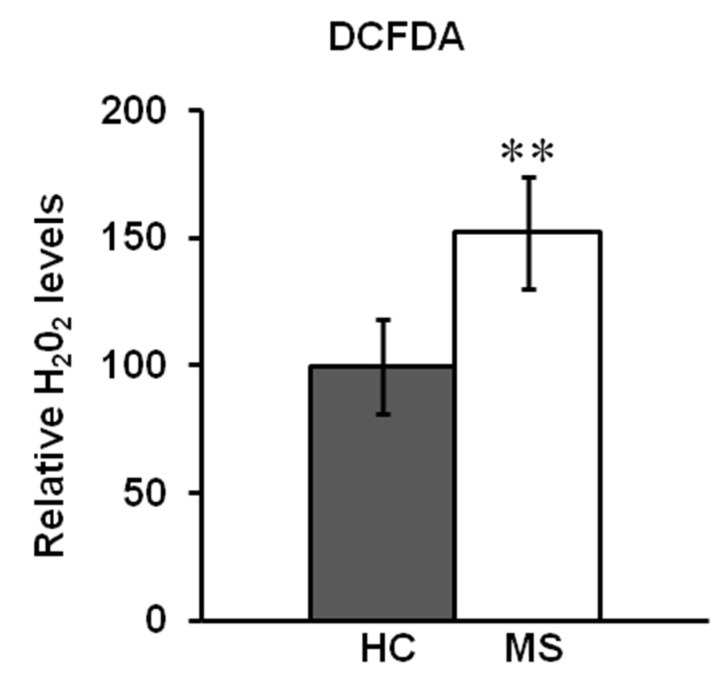
H_2_O_2_ production in PBMC of HC and MS patients. H_2_O_2_ level was detected spectrofluorimetrically by dichlorodihydrofluorescin (DCFDA) probe. The mean of HC was set to 100% and the mean value of MS expressed as percentage of intensity of fluorescence respect to HC. The histograms represent the means of values ± SEM. (** *p* < 0.01; Student’s *t*-test).

Processing of OPA1 is regulated by different proteins and cellular conditions such as oxidative stress. Measurement of H_2_O_2_ level, detected by the redox-sensitive fluorescent probe DCFDA, showed increased ROS production in the PBMCs of MS patients compared to HC (Figure 2).

**Figure 3:**
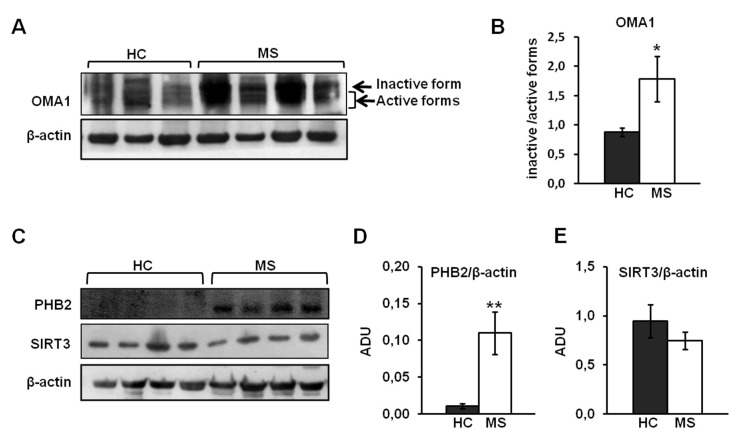
OMA1, SIRT3, and PHB2 in PBMCs of HC and MS patients. (**A**,**C**) Representative images of Western blotting analysis. Proteins from PBMC from HC and MS were loaded on 7.5% SDS-PAGE. After separation, the proteins were transferred on nitrocellulose membranes and immunoblotted with the antibodies against OMA1, SIRT3, and PHB2. Protein loading was assessed with β-actin antibody. The immunoblotting against β-actin in panel C is the same shown in the Figure 1A, belonging to the same experiment series (**B**,**D**). (**B**) The histograms represent the means of ratio values ± SEM between the ADU of inactive and active forms of OMA1 in HC and MS subjects. (**D**,**E**) The ADU of PHB2 and SIRT3 were normalized to ADU of β-actin. The histograms represent the means of values of ADU ± SEM of samples. (* *p* < 0.05, ** *p* < 0.01; Student’s *t*-test).

This result prompted to investigate on stress regulated protein OMA1, PHB2 and SIRT3 that are involved in OPA1 processing and stabilization [20,22,25]. Activation of OMA1 protease is accompanied by its autocatalytic degradation that results in the complete turnover of protein [22]. Western blotting analysis of OMA1 did not show the activation of this protease in PBMCs from MS samples as revealed by the increased ratio between inactive and active forms (Figure 3A,B). We next examined PHB2 level by western blot analysis with specific antibody. An increased PHB2 protein level was observed in MS patients (Figure 3C,D) compared to HC samples, while no difference was observed for SIRT3 protein level (Figure 3C,D). 

**Figure 4:**
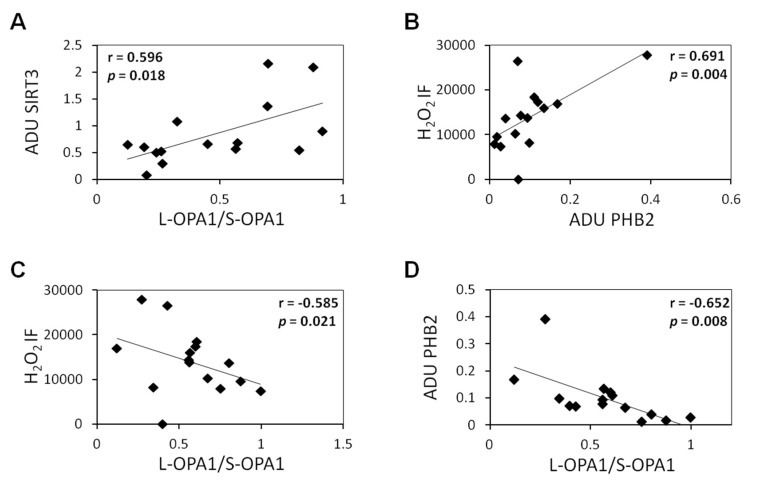
Correlation plots. Empty squares indicates HC group, full squares indicates MS group (**A**)Scatter plot and linear regression of data of relationship between SIRT3 protein level, expressed in ADU, and L-OPA1/S-OPA1 balance in HC group (correlation coefficient, r = 0.596; *P*, 0.021). (**B**) Scatter plot and linear regression of data of relationship between H_2_O_2_, expressed in intensity of fluorescence (IF), and PHB2 protein level, expressed in ADU in MS group (correlation coefficient, r = −0.691; *P*, 0.004). (**C**) Scatter plot and linear regression of data of relationship between H_2_O_2_ and L-OPA1/S-OPA1 balance in MS group (correlation coefficient r = −0.585, *P*,0.021). (**D**) Scatter plot and linear regression of data of relationship between PHB2 protein expression and cleaved long OPA1 (L-OPA1)/cleaved short OPA1 (S-OPA1) balance in MS group (correlation coefficient, r = −0.652; *P* = 0.008). Degree of freedom 13.

To explore whether the alterations in the analyzed molecular parameters can cross correlate with each other, a correlation analysis was performed for HC and MS groups. The results in HC indicated a remarkable positive correlation of SIRT3 changes with changes of L- and S-OPA1 balance. This correlation was lost in MS group. In addition, in MS group, a significant positive correlation was observed between H_2_O_2_ level and PHB2 (Figure 4B), and negative correlations between H_2_O_2_ and L- and S-OPA1 balance (Figure 4C) and between PHB2 and L- and S-OPA1 balance (Figure 4D).

## 4. Discussion

The pathogenesis of MS involves autoreactive T lymphocytes that have the capacity to invade the CNS causing demyelization and axonal damage [4]. The deletion of autoreactive lymphocytes is normally mediated by apoptosis, however, an escape from mitochondria-mediated apoptosis has been reported in lymphocytes of MS patients [12]. Mitochondria from MS lymphocytes also show a decrease of mitochondrial respiration [13] associated with a specific decrease of complex I and complex IV activities [28], and, of note, mitochondria have been found to range in shape and size and showed thickened cristae [29]. Mitochondrial dependent apoptosis is also depending on mitochondrial respiration, shape and structure. A growing body of evidence suggests that OPA1 participates through several mechanisms in defining mitochondrial shape and structure of cristae [14] and modulating mitochondrial respiration [17]. This modulates cell susceptibility towards apoptotic stimuli [14]. Augmented level of OPA1, formation of OPA1 oligomers and a correct balance of L and S forms are considered anti-apoptotic factors [17]. In light of this and the data reporting that OPA1 mutations are associated with multiple sclerosis-like symptoms [18], in this work, we analyzed OPA1 and its modulators in PBMCs of MS patients compared to HC. Fifteen healthy controls and fifteen subjects affected by MS were enrolled in the study. The patients group included 12 females and 3 males and 11 RR forms and 4 SP forms of MS (see Table 1). No significant difference was observed between males and females subjects as well as between RR and SP forms.

Our results showed the same level of OPA1 total protein (L+S) in HC and SM samples and no differences were observed in the balance between L and S forms of OPA1 in PBMC of HC and MS patients. Interesting the analysis of the electrophoretic migration of immune-revealed bands of OPA1 in lymphocytes of MS samples showed only one S-form at a higher molecular weight in MS with respect to the two S forms observed in HC, thus suggesting a characteristic processing of OPA1 in MS samples. The functions of OPA1 are regulated, under stress conditions, by OMA1-dependent proteolytic cleavage. As expected PBMCs of MS patients show increased level of ROS that, anyway, not results in the activation of stress-induced OMA1 [22], as shown by the accumulation of inactive form of OMA1, thus suggesting an adaptation to exposure at chronic OS. Of note, it was reported that suppression of OMA1 activity strongly prevented cytochrome *c* release into the cytosol [30] and cells lacking the protease OMA1 showed an increased resistance to external apoptotic stimuli [31,32]. OPA1, beyond the proteolytic control, is also under the control of alternative splicing. Eight different OPA1 isoforms, generated by alternative splicing of four exons near the amino terminus (Figure S1), are characterized for the presence, or absence, of at least three different proteolytic cleavage sites, named S1, S2, and S3 [33]. The S1 site is cleaved by OMA1 while S2 and S3 by Ymel1 protease. The presence of isoform 3, which contains S3 and S1 cleavage sites (see Figure S1), together with the inactivation of OMA1 protease (see also [32]), could explain the shift of electrophoretic mobility observed in MS patients. Although YME1L and OMA1 constitutively control OPA1, the proteolytic processing of OPA1 is more complex, in fact, other proteases can act on OPA1 under particular stress condition or metabolic demands [33,34], thus, the involvement of other mitochondrial proteases cannot be ruled out. In this contest, should be noted that, in MS, several proteases are involved [35,36] and that mitochondrial proteases are often involved in the pathogenesis of neurological diseases [37].

It has been reported that SIRT3 has an important role in a variety ofoxidative stress-mediated cellular responses [38], in the regulation of bioenergetic function and antioxidant defense of mitochondria under OS conditions [39]. OS regulated SIRT3 protein level that, in turn, is involved in mitochondrial apoptosis by modulating OPA1 acetylation/processing [20]. In particular, sustained level of SIRT3 protein favors the apoptosis resistance, while a decrease promotes cell death [20]. We have also investigated on SIRT3 protein level; however, despite the increased ROS production in MS, no difference has been observed in MS lymphocytes compared to HC samples. Although the SIRT3 protein level was unchanged, this could be interpreted as a loss of response of lymphocytes of MS patients to OS. The data on SIRT3 and OMA1 suggest a deregulation of normally stress response mechanisms of these proteins in PBMCs of MS patients.

Furthermore, OPA1 processing and stability is also controlled by PHB2 protein. In mitochondrial inner membrane, PHB2 protein forms with PHB1 a large membrane-bound complex [40,41]. This complex is required for OPA1 stability [42], indeed the deletion of PHB2 leads to the impaired cellular proliferation, aberrant mitochondrial cristae morphogenesis, and apoptosis [24,42], while an over-expression of PHB2 is reported to protect cells from apoptosis [43]. We show, according with others [26] and as response to OS [25], a strong increase of PHB2 protein level in MS samples, thus representing another element of resistance to apoptosis. It worth mentioning that an elevated autophagic flux has been reported in autoreactive T cells, both in patients and in the mouse model of experimental autoimmune encephalomyelitis [44]. It has been found that PHB2 participates in the mitophagy process (selective autophagy) by functioning as mitochondrial receptor in autophagosome formation [45].

Interestingly, using Pearson’s correlation analysis, we found a positive correlation in HC group between SIRT3 changes and changes of L- and S-OPA1 balance. This is in agreement with the findings showing that the SIRT3-dependent deacetylation of L-OPA1 inhibits its proteolytic processing to form S-OPA1 [20]. This correlation was lost in the MS group, while changes of L- and S-OPA1 balance, PHB2 protein level and H_2_O_2_ correlated each other. In facts, under condition of oxidative stress, an increased cleavage of L-OPA1 to produce S-OPA1 has been observed [20] as well as an increase of PHB2 expression [25]. The over expression of PHB2 has been found to protect the cell from oxidative stress-dependent apoptosis [43].

## 5. Conclusions

Our data showed that, in PBMCs of MS patients, oxidative stress is associated with increased level of PHB2 and stabilization of OMA1. We propose that the alteration of PHB2, OMA1, and OPA1 is involved in the apoptosis resistance. We hypothesize involvement of specific proteolytic processing of OPA1 in lymphocytes of MS patients. Other investigations will be needed to define the mechanisms at the bases of this deregulations and the possible proteases involved in the characteristic processing of OPA1 in MS samples. The specific OPA1 electrophoretic profile and an increased level of PHB2 in MS patients could also be taken in account to assess disease activity. Understanding of the molecular mechanisms underlying these deregulations could shed light on new therapeutic interventions.

## Figures and Tables

**Table 1:** Demographic and clinical characteristics of patients with multiple sclerosis (MS) and healthy control subjects (HC) enrolled into the study. (SP: secondary progressive, RR: relapsing-remitting, EDSS: Expanded Disability Status Scale, SEM: standard error of mean.

	MS	HC
Subject (number)	15	15
Age (year)	45 ± 2.46 SEM	44.92 ± 3.92 SEM
Gender	12 Females	6 Females
	3 Males	9 Males
MS form	11 RR	
	4 SP	
Disease duration(year)	14.4 ± 1.70 SEM	
EDSS	4.2 ± 0.34 SEM	

## Supplementary Materials

The following are available online at https://www.mdpi.com/2227-9059/8/4/85/s1.

## References

[1] Tobore T.O. (2020). Towards a comprehensive etiopathogenetic and pathophysiological theory of multiple sclerosis. Int. J. Neurosci..

[2] Lassmann H., Brück W., Lucchinetti C.F. (2007). The immunopathology of multiple sclerosis: An overview. Brain Pathol..

[3] Loma I., Heyman R. (2011). Multiple sclerosis: Pathogenesis and treatment. Curr. Neuropharmacol..

[4] Sospedra M., Martin R. (2005). Immunology of multiple sclerosis. Annu. Rev. Immunol..

[5] Gonzalo H., Nogueras L., Gil-Sánchez A., Hervás J.V., Valcheva P., González-Mingot C., Martin-Gari M., Canudes M., Peralta S., Solana M.J. (2019). Impairment of Mitochondrial Redox Status in Peripheral Lymphocytes of Multiple Sclerosis Patients. Front. Neurosci..

[6] Gilgun-Sherki Y., Melamed E., Offen D. (2004). The role of oxidative stress in the pathogenesis of multiple sclerosis: The need for effective antioxidant therapy. J. Neurol..

[7] Ohl K., Tenbrock K., Kipp M. (2016). Oxidative stress in multiple sclerosis: Central and peripheral mode of action. Exp. Neurol..

[8] Comabella M., Khoury S.J. (2012). Immunopathogenesis of multiple sclerosis. Clin. Immunol..

[9] McFarland H.F., Martin R. (2007). Multiple sclerosis: A complicated picture of autoimmunity. Nat. Immunol..

[10] Segal B.M., Cross A.H. (2000). Fas(t) track to apoptosis in MS: TNF receptors may suppress or potentiate CNS demyelination. Neurology.

[11] Ruggieri M., Avolio C., Scacco S., Pica C., Lia A., Zimatore G.B., Papa S., Livrea P., Trojano M. (2006). Glatiramer acetate induces pro-apoptotic mechanisms involving Bcl-2, Bax and Cyt-c in peripheral lymphocytes from multiple sclerosis patients. J. Neurol..

[12] Julià E., Edo M.C., Horga A., Montalban X., Comabella M. (2009). Differential susceptibility to apoptosis of CD4+T cells expressing CCR5 and CXCR3 in patients with MS. Clin. Immunol..

[13] La Rocca C., Carbone F., De Rosa V., Colamatteo A., Galgani M., Perna F., Lanzillo R., Brescia Morra V., Orefice G., Cerillo I. (2017). Immunometabolic profiling of T cells from patients with relapsing-remitting multiple sclerosis reveals an impairment in glycolysis and mitochondrial respiration. Metabolism.

[14] Benard G., Rossignol R. (2008). Ultrastructure of the mitochondrion and its bearing on function and bioenergetics. Antioxid. Redox Signal..

[15] Kalkavan H., Green D.R. (2018). MOMP, cell suicide as a BCL-2 family business. Cell Death Differ..

[16] Wai T., Langer T. (2016). Mitochondrial Dynamics and Metabolic Regulation. Trends Endocrinol. Metab..

[17] Pernas L., Scorrano L. (2016). Mito-Morphosis: Mitochondrial Fusion, Fission, and Cristae Remodeling as Key Mediators of Cellular Function. Annu. Rev. Physiol..

[18] Yu-Wai-Man P., Spyropoulos A., Duncan H.J., Guadagno J.V., Chinnery P.F. (2016). A multiple sclerosis-like disorder in patients with OPA1 mutations. Ann. Clin. Trans. Neurol..

[19] Patten D.A., Wong J., Khacho M., Soubannier V., Mailloux R.J., Pilon-Larose K., MacLaurin J.G., Park D.S., McBride H.M., Trinkle-Mulcahy L. (2014). OPA1-dependent cristae modulation is essential for cellular adaptation to metabolic demand. EMBO J..

[20] Signorile A., Santeramo A., Tamma G., Pellegrino T., D’Oria S., Lattanzio P., De Rasmo D. (2017). Mitochondrial cAMP prevents apoptosis modulating Sirt3 protein level and OPA1 processing in cardiac myoblast cells. Biochim. Biophys. Acta Mol. Cell. Res..

[21] MacVicar T., Langer T. (2016). OPA1 processing in cell death and disease—The long and short of it. J. Cell Sci..

[22] Baker M.J., Lampe P.A., Stojanovski D., Korwitz A., Anand R., Tatsuta T., Langer T. (2014). Stress-induced OMA1 activation and autocatalytic turnover regulate OPA1-dependent mitochondrial dynamics. EMBO J..

[23] Rainbolt T.K., Lebeau J., Puchades C., Wiseman R.L. (2016). Reciprocal Degradation of YME1L and OMA1 Adapts Mitochondrial Proteolytic Activity during Stress. Cell Rep..

[24] Merkwirth C., Dargazanli S., Tatsuta T., Geimer S., Löwer B., Wunderlich F.T., von Kleist-Retzow J.C., Waisman A., Westermann B., Langer T. (2008). Prohibitins control cell proliferation and apoptosis by regulating OPA1-dependent cristae morphogenesis in mitochondria. Genes Dev..

[25] Ross J.A., Robles-Escajeda E., Oaxaca D.M., Padilla D.L., Kirken R.A. (2017). The prohibitin protein complex promotes mitochondrial stabilization and cell survival in hematologic malignancies. Oncotarget.

[26] Kumar M.K.S., Nair S., Mony U., Kalingavarman S., Venkat R., Sivanarayanan T.B., Unni A.K.K., Rajeshkannan R., Anandakuttan A., Radhakrishnan S. (2018). Significance of elevated Prohibitin 1 levels in Multiple Sclerosis patients lymphocytes towards the assessment of subclinical disease activity and its role in the central nervous system pathology of disease. Int. J. Biol. Macromol..

[27] Polman C.H., Reingold S.C., Banwell B., Clanet M., Cohen J.A., Filippi M., Fujihara K., Havrdova E., Hutchinson M., Kappos L. (2011). Diagnostic criteria for multiple sclerosis: 2010 revisions to the McDonaldcriteria. Ann. Neurol..

[28] De Riccardis L., Rizzello A., Ferramosca A., Urso E., De Robertis F., Danieli A., Giudetti A.M., Trianni G., Zara V., Maffia M. (2015). Bioenergetics profile of CD4+T cells in relapsing remittingmultiple sclerosissubjects. J. Biotechnol..

[29] Djaldetti R., Achiron A., Ziv I., Djaldetti M. (1995). Lymphocyte ultrastructure in patients with multiple sclerosis. Biomed. Pharmacother..

[30] Jiang X., Jiang H., Shen Z., Wang X. (2014). Activation of mitochondrial protease OMA1 by Bax and Bak promotes cytochrome c release during apoptosis. Proc. Natl. Acad. Sci. USA.

[31] Anand R., Wai T., Baker M.J., Kladt N., Schauss A.C., Rugarli E., Langer T. (2014). The i-AAA protease YME1L and OMA1 cleave OPA1 to balance mitochondrial fusion and fission. J. Cell Biol..

[32] Quirós P.M., Ramsay A.J., Sala D., Fernández-Vizarra E., Rodríguez F., Peinado J.R., Fernández-García M.S., Vega J.A., Enríquez J.A., Zorzano A. (2012). Loss of mitochondrial proteaseOMA1alters processing of the GTPase OPA1 and causes obesity and defective thermogenesis in mice. EMBO J..

[33] Van der Bliek A.M., Shen Q., Kawajiri S. (2013). Mechanisms of mitochondrial fission and fusion. Cold Spring Harb. Perspect. Biol..

[34] Sood A., Jeyaraju D.V., Prudent J., Caron A., Lemieux P., McBride H.M., Laplante M., Tóth K., Pellegrini L. (2014). A Mitofusin-2-dependent inactivating cleavage of Opa1 links changes in mitochondria cristae and ER contacts in the postprandial liver. Proc. Natl. Acad. Sci. USA.

[35] Scarisbrick I.A. (2008). The multiple sclerosis degradome: Enzymatic cascades in development and progression of central nervous system inflammatory disease. Advances in Multiple Sclerosis and Experimental Demyelinating Diseases.

[36] Muri L., Leppert D., Grandgirard D., Leib S.L. (2019). MMPs and ADAMs in neurological infectious diseases and multiple sclerosis. Cell. Mol. Life Sci..

[37] Kozin M.S., Kulakova O.G., Favorova O.O. (2018). Involvement of Mitochondria in Neurodegeneration in Multiple Sclerosis. Biochemistry.

[38] Singh C.K., Chhabra G., Ndiaye M.A., Garcia-Peterson L.M., Mack N.J., Ahmad N. (2018). The Role of Sirtuins in Antioxidant and Redox Signaling. Antioxid. Redox Signal..

[39] Wu Y.T., Wu S.B., Wei Y.H. (2014). Roles of sirtuins in the regulation of antioxidant defense and bioenergetic function of mitochondria under oxidative stress. Free Radic. Res..

[40] Nijtmans L.G., Artal S.M., Grivell L.A., Coates P.J. (2002). The mitochondrial PHB complex: Roles in mitochondrial respiratory complex assembly, ageing and degenerative disease. Cell. Mol. Life Sci..

[41] Signorile A., Sgaramella G., Bellomo F., De Rasmo D. (2019). Prohibitins: A Critical Role in Mitochondrial Functions and Implication in Diseases. Cells.

[42] Merkwirth C., Langer T. (2009). Prohibitin function within mitochondria: Essential roles for cell proliferation and cristae morphogenesis. Biochim. Biophys. Acta.

[43] Muraguchi T., Kawawa A., Kubota S. (2010). Prohibitin protects against hypoxia-induced H9c2 cardiomyocyte cell death. Biomed. Res..

[44] Alirezaei M., Fox H.S., Flynn C.T., Moore C.S., Hebb A.L., Frausto R.F., Bhan V., Kiosses W.B., Whitton J.L., Robertson G.S. (2009). Elevated ATG5 expression in autoimmune demyelination and multiple sclerosis. Autophagy.

[45] Wei Y., Chiang W.C., Sumpter R., Mishra P., Levine B. (2017). Prohibitin 2 Is an Inner Mitochondrial Membrane Mitophagy Receptor. Cell.

