# Supplementary material for: PBMC of Multiple Sclerosis Patients Show Deregulation of OPA1 Processing Associated with Increased ROS and PHB2 Protein Levels
Source: Biomedicines. 2020 Apr 11;8(4):85. doi: 10.3390/biomedicines8040085 (PMC7235786; doi:10.3390/biomedicines8040085)
Supplement: Supplementary file 1 [file biomedicines-08-00085-s001.pdf]

Table S1 Western blotting images were analyzed by Image Lab Touch 2.4 software (BioRAD) for calculation, in each lane, of Relative front (Rf) of L-OPA1 and S-OPA1 bands. "d" represents the difference between Rf of S-OPA1 and Rf of L-OPA1 bands in each lane. The table reports single value of each sample and the means values ± SEM of HC and MS samples. (p values are calculated by Student's t-test).

	**Rf L-OPA1 band**	**Rf S-OPA1 band**	**d**
HC1	0,3090	0,4452	0,1362
HC2	0,2757	0,4319	0,1561
HC3	0,2625	0,4020	0,1395
HC4	0,2757	0,3953	0,1196
HC5	0,2236	0,3389	0,1154
HC6	0,3436	0,5084	0,1648
HC7	0,3520	0,4497	0,0978
HC8	0,3395	0,4648	0,1254
HC9	0,3232	0,4238	0,1006
HC10	0,3395	0,4648	0,1254
HC11	0,3272	0,4251	0,0979
HC12	0,3154	0,4609	0,1456
HC13	0,3450	0,4394	0,0943
HC14	0,3370	0,4558	0,1188
HC15	0,3094	0,4890	0,1796
**Mean HC**	**0,3119**	**0,4397**	**0,1278**
SEM	0,0095	0,0105	0,0067
MS1	0,2658	0,3621	0,0963
MS2	0,2791	0,3588	0,0797
MS3	0,2791	0,3821	0,1030
MS4	0,2924	0,3721	0,0797
MS5	0,2236	0,2885	0,0649
MS6	0,2163	0,2933	0,0769
MS7	0,2308	0,2933	0,0625
MS8	0,2236	0,2981	0,0745
MS9	0,3603	0,4553	0,0950
MS10	0,3631	0,4665	0,1034
MS11	0,3313	0,4255	0,0942
MS12	0,3058	0,4067	0,1009
MS13	0,2844	0,3853	0,1009
MS14	0,3423	0,4286	0,0863
MS15	0,3585	0,4609	0,1024
**Mean MS**	**0,2904**	**0,3785**	**0,0880**
SEM	0,0135	0,0163	0,0036
p	0,2051	0,0038	0,000015

Supplementary figure 1 (S1)

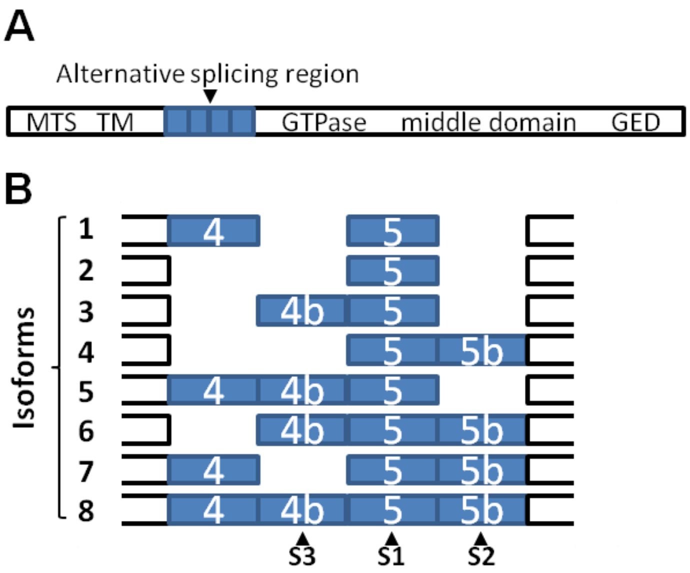

Figure S1. A, Schematic representation of the alternative splicing region (blue boxes) in protein domains of OPA1. MTS: Mitochondrial targeting sequence ; TM: transmembrane segment, GED: GTPase effector domain. B, Alternatively spliced exons (exon 4, exon 4b, exon 5 and exon 5b) generate eight isoforms with the presence or absence of cleavage sites (S1, S2 and S3). S2 and S3 sites are constitutively cleaved by YME1L while S1 is cleaved by OMA1.

Supplementary figure 2 (S2)

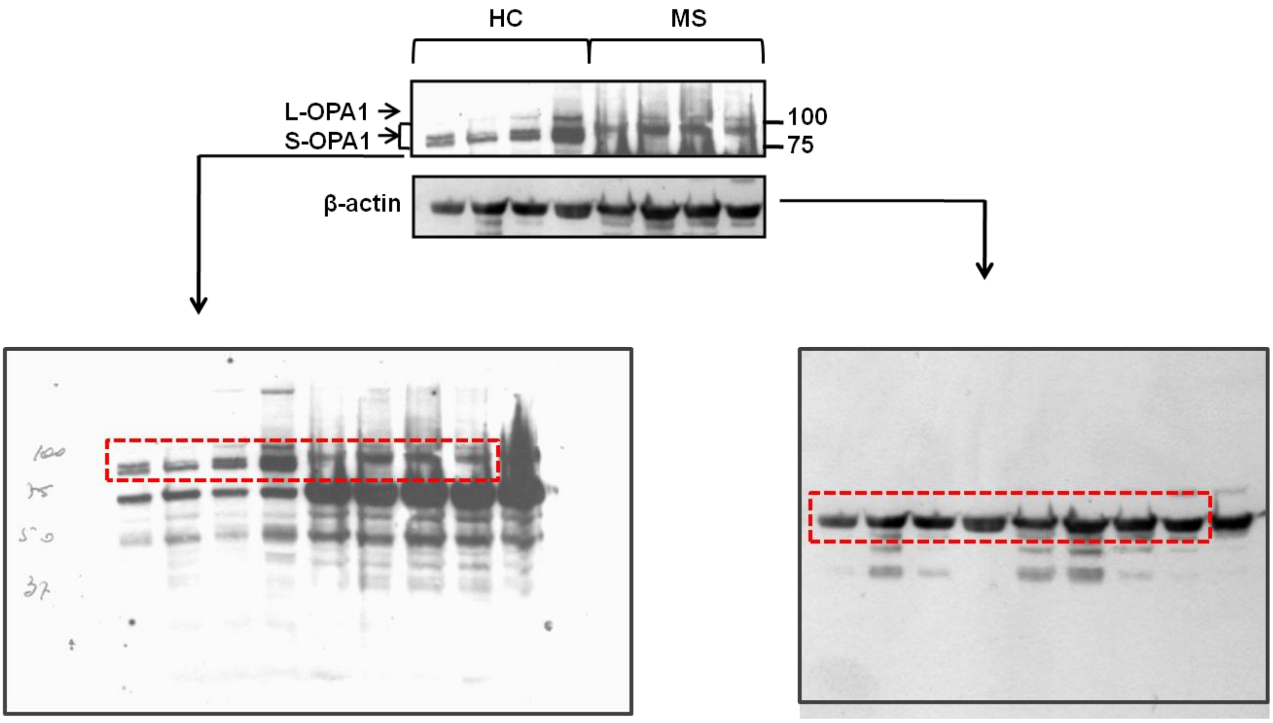

Figure S2 Full original images of western blots of OPA1 and β-actin shown in Figure 1, panel A. HC: healthy control subjects; MS: multiple sclerosis patients. Cropped areas are marked by red color.

Supplementary figure 3 (S3)

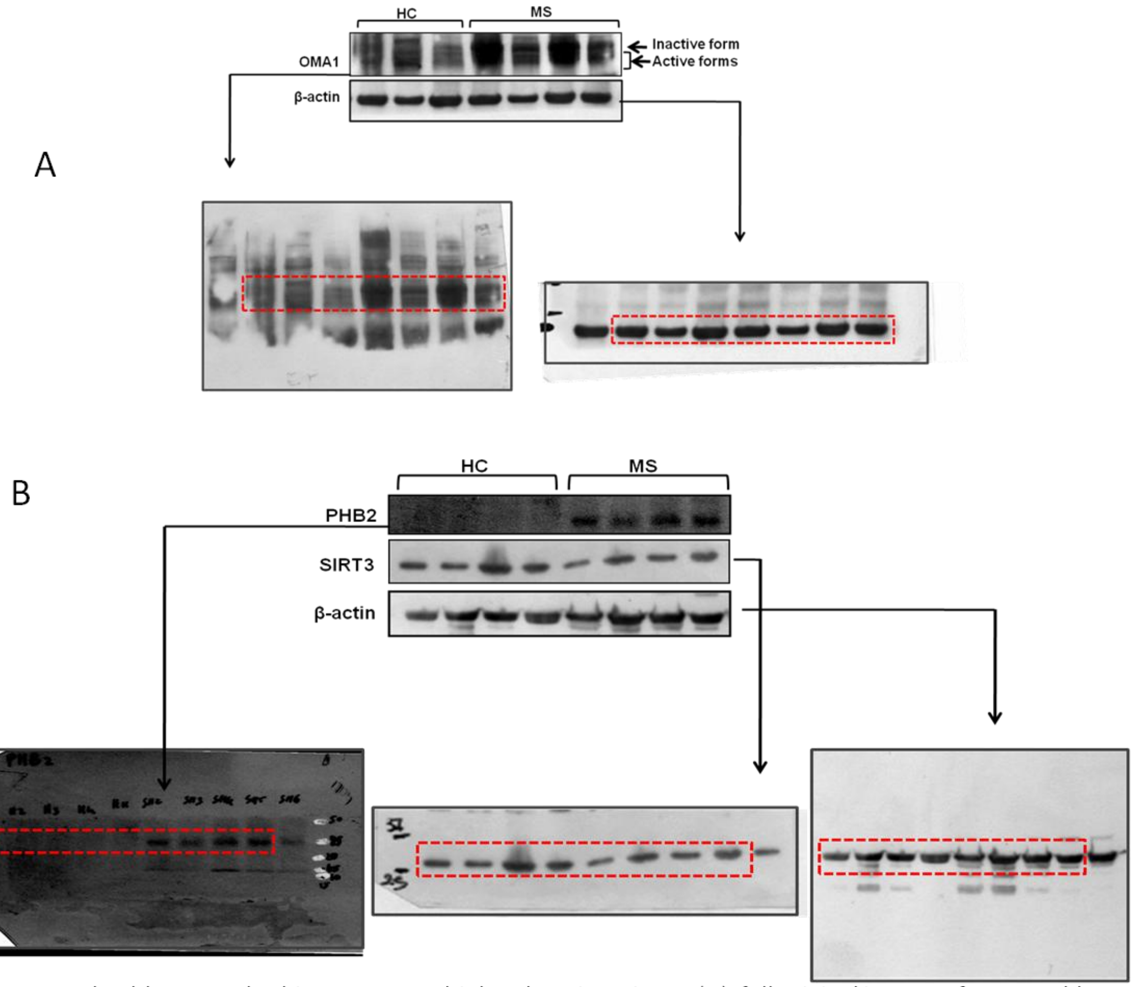

Figure S3 HC: healthy control subjects; MS : multiple sclerosis patients. (A) full original images of western blots of OMA1 and β-actin shown in Figure 3, panel A. (B) full original images of western blots of PHB2, SIRT3 and β-actin shown in Figure 3, panel C. Cropped areas are marked by red color.
